# Diverse *dif* module content and configurations in the r3-T5 group of Rep_3/OrfX plasmids from *Acinetobacter* species reveal extensive *dif* module shuffling

**DOI:** 10.1128/spectrum.03186-25

**Published:** 2026-03-24

**Authors:** Stephanie J. Ambrose, Shelly Lingxuan Xiao, Ruth M. Hall

**Affiliations:** 1 School of Life and Environmental Sciences, The University of Sydney 98483, Sydney, New South Wales, Australia; 2 Sydney Infectious Diseases Institute, The University of Sydney 442308 , Sydney, New South Wales, Australia; Shenzhen University School of Medicine, Shenzhen, China

**Keywords:** Rep_3/OrfX plasmid, p*dif *site, *dif *module, *Acinetobacter *species plasmids

## Abstract

The *dif* module mobile element system is found naturally in *Acinetobacter* plasmids with a backbone that encodes a Rep_3 replication initiation function and an OrfX open reading frame. Here, the characteristics of plasmid pMSHR_A204 (10,025 bp) from the r3-T5 subgroup of this family and related plasmids were examined. pMSHR_A204 consists of a 3.27 kbp backbone C module and five accessory *dif* modules (two C and three D modules), including the *tet39* tetracycline resistance module and a large fusion module carrying *higBA2* toxin-antitoxin genes. Rearrangements of the *dif* module content, both inversions and deletions involving p*dif* sites with identical or nearly identical spacers, were detected experimentally. Sequences of 35 unique r3-T5 plasmids retrieved from GenBank and originating from three different *Acinetobacter* species and 27 countries were annotated and compared. The backbone C module was highly conserved but was associated with a variety of accessory *dif* modules. Most plasmids carried a *dif* module containing toxin-antitoxin genes. Eleven plasmids were cointegrates with a second backbone from another r3 type. Most non-cointegrate r3-T5 plasmids carried odd numbers of *dif* modules (e.g., 2 D modules and 1 C module), but several included a fusion (CD) module and two or four *dif* modules. Varied configurations caused by reactions between p*dif* sites with identical or nearly identical spacers were detected within groups of plasmids with the same overall *dif* module content. One pair differed by a single *dif* module. These rearrangements were consistent with models for movement of *dif* modules between plasmids, including via a cointegrate intermediate.

**IMPORTANCE:**

The plasmids that are associated with *Acinetobacter* isolates appear to be restricted to this genus. However, the unique properties of the many and various plasmid types found in *Acinetobacter* species have received little attention relative to the plasmids of other gram-negative pathogens. In addition, the novel *dif* module mobile element system, also found uniquely in *Acinetobacter* species, was recently shown to be naturally associated with a specific type of *Acinetobacter* plasmid. To investigate the dynamics of *dif* module movement, here, the properties and configurations of one group of plasmids with identical or closely related backbones that carry different *dif* modules or the same modules in different configurations have been examined. This will increase understanding of the global distribution and diversity of plasmids that carry *dif* modules, as well as provide insight into the way *dif* modules relocate.

## INTRODUCTION

Genome sequencing has revealed that a very large number of plasmid types are found in *Acinetobacter baumannii* (1) and other *Acinetobacter* species (2). However, *Acinetobacter* plasmid types are different from those found in other gram-negative species, and they are not found in Enterobacteriaceae. Having been clearly identified only relatively recently, they are not as well characterized as the plasmids of gram-positive and gram-negative species that have been studied for many decades. One very large group of plasmids harbored by *Acinetobacter* species has unique properties, being made up of a backbone module that contains a *repA* gene encoding a Rep_3 replication initiation protein and a highly variable accessory segment made up of at least one discrete module known as a *dif* module (3–6). The unusual properties of these plasmids were first noticed because a few *dif* modules harbor an antibiotic resistance gene (5, 7, 8); however, genes found in *dif* modules identified subsequently encode a variety of proteins with diverse functions, such as several toxin-antitoxin pairs (3, 5), as well as a serine recombinase, chromate and hydrogen peroxide resistance determinants, an alcohol dehydrogenase, and a sulfate permease (3, 5, 9–15). *Acinetobacter* species are widespread in the environment (16), and two of the antibiotic resistance genes found in *dif* modules, namely the *tet39* tetracycline resistance module and the *msrE-mphE* macrolide resistance module (5), are among the most abundant resistance genes found in environmental samples (17–19).

The majority of *Acinetobacter* plasmids can be classified using the sequence of the *repA* gene. In the most recent classification scheme (1), plasmids carrying a *repA* gene are first separated into groups of those that encode Rep_1, Rep_3, or RepPriCT type replication initiation proteins; then those with less than 5% difference in the *repA* gene are grouped into subtypes, for example, r3-T1, r3-T2, etc. The plasmids that naturally include at least one *dif* module encode a Rep_3 family replication initiation protein, and a recent analysis of representatives of 78 types in the Rep_3 group revealed that nearly half of them consist of a backbone module and at least one *dif* module (4). These plasmids have been designated Rep_3/OrfX plasmids because, in addition to a *repA* gene and upstream iterons, the backbone module characteristically includes an orf downstream of *repA* that encodes a homolog of a protein of unknown function, designated orfX/OrfX, that may be involved in *dif* module movement (4).

The *dif* modules associated with the Rep_3/OrfX plasmids are separated from each other and from the backbone by p*dif* sites that resemble the *dif* site found near the terminus of bacterial chromosomes (5, 7, 12, 20, 21), and it has been widely assumed that they are mobilized by the XerC and XerD site-specific recombinases. XerC and XerD act together to recombine the *dif* sites in chromosomal dimers, thereby ensuring chromosome separation for partition into daughter cells (22, 23). XerC and XerD purified from *A. baumannii* have been shown to bind to p*dif* sites as well as the *A. baumannii dif* site (24, 25) and recently, a requirement for the *A. baumannii* XerC for recombination between a pair of p*dif* sites *in vivo* has been demonstrated (24).

**Fig 1:**
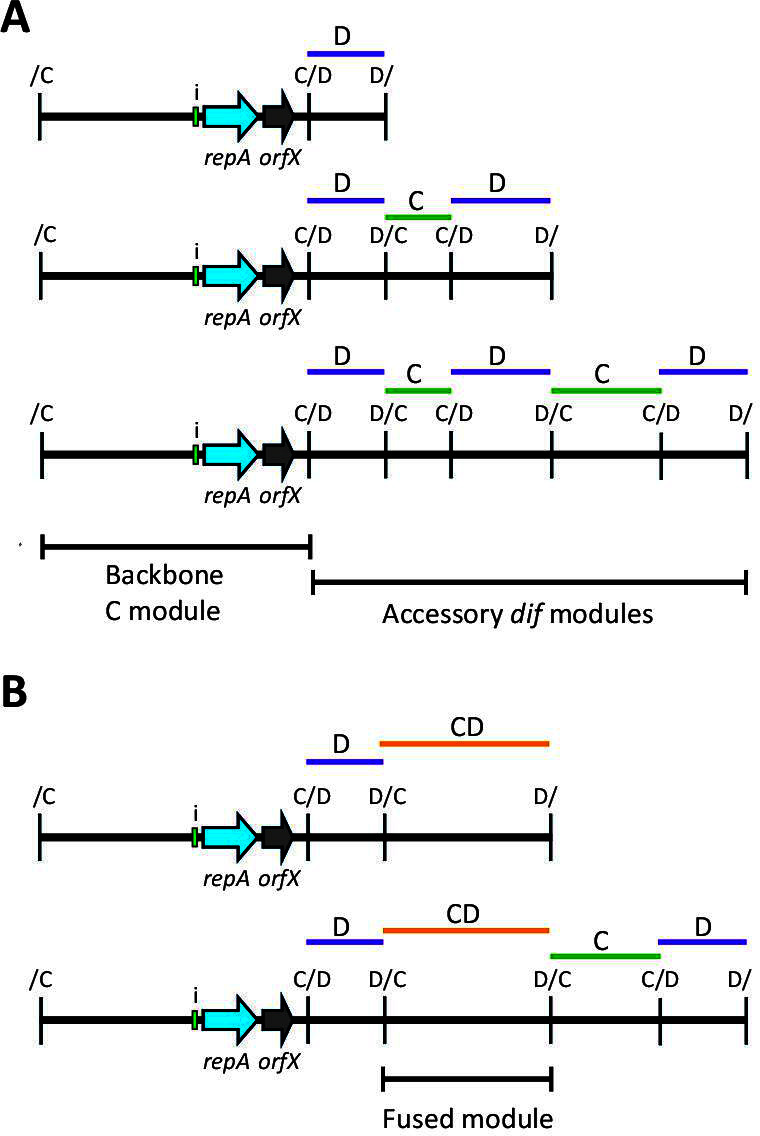
Schematic of the structure of Rep_3/OrfX plasmids carrying *dif* modules in (**A**) the standard *dif* module configuration and (**B**) carrying a fused *dif* module. Arrows indicate the extent and orientation of *repA* (light blue) and orfX (gray), with their names below. The iteron region in each plasmid is indicated by a green box. The p*dif* sites are indicated by a vertical bar, with the orientation of the site (C/D or D/C) indicated above. Modules with C sites internal (C modules) or D sites internal (D modules) are indicated above by green or purple bars, respectively, labeled C or D. In panel **A**, the backbone C module region and the accessory *dif* module region are indicated by horizontal bars below. In panel **B**, the module that has one C site and one D site internal (CD module) is indicated by an orange bar above and labeled CD. The extent of the fused module is also indicated by a horizontal bar below.

The backbones of Rep_3/OrfX plasmids have a p*dif* site at each end and hence resemble *dif* modules but are larger than the average accessory *dif* module (4). Each p*dif* site, like the chromosomal *dif* site, is made up of a binding site for the XerC site-specific recombinase and one for the XerD recombinase, separated by a 6 bp spacer. Because the XerC and XerD binding sites are distinct, the p*dif* sites harbor intrinsic orientation information. One consequence of this asymmetry is that the *dif* modules come in two configurations, those with the XerC sites internal, known as C modules, and D modules with XerD sites internal. To generate complete p*dif* sites, C and D modules must alternate and, as the inversely oriented p*dif* sites surrounding the plasmid backbone make it a C module, a viable plasmid is formed only when it is combined with a D module (3, 4). Thereafter, *dif* modules must be added in pairs consisting of one C and one D module, as shown in Fig. 1A. The exception is where two or more modules have become fused to generate a CD module surrounded by two C/D or two D/C sites (Fig. 1B), and how these CD modules arise has been documented in a few cases (3, 10).

Plasmids in the Rep_3/OrfX group are known to form cointegrates that include two different *rep* genes encoding two different RepA replication initiation proteins. An example is found in pACICU2, one of the earliest sequenced *Acinetobacter* plasmids, which carries both an r3-T1 and r3-T19 (formerly Aci1 and Aci10) *repA* gene, and the same combination is seen in pA388 (6). Other examples of cointegrates have been reported (3, 10, 14, 21, 26, 27). As noted by Balalovski and Grainge (20), cointegrates have the potential to act as intermediates that enable the relocation of *dif* modules, and formation and resolution of a cointegrate made up of two compatible Rep_3 plasmids present in the same cell via recombination at a pair of p*dif* sites with identical spacer sequences has been demonstrated in one case (21, 27).

Here, we have examined the properties of a plasmid found in a tetracycline-resistant clinical *A. baumannii* isolate MSHR_A204 (ST10:KL108:OCL2) that was acquired in the community and is believed to have been acquired by the patient from the environment (28). The draft genome of this isolate was known to include only the *tet39* tetracycline resistance determinant, and here, a plasmid designated pMSHR_A204 was assembled from available short-read data. Rearrangements of the *dif* modules in pMSHR_A204 were identified experimentally. To begin to systematically explore the properties of plasmids that carry *dif* modules, the complete sequences of the group of plasmids that include the same *repA* gene as pMSHR_A204 were retrieved from the GenBank non-redundant database together with associated metadata. For each unique plasmid configuration, p*dif* sites and protein-coding regions were identified and annotated, and the backbones compared. Plasmids with the same or similar *dif* module content but different organizations were examined to determine how rearrangements occurred and deduce the features of p*dif* sites associated with inversion, deletion/excision, and movement of one or more *dif* modules from one plasmid to another.

## RESULTS

### Structure of two forms of plasmid pMSHR_A204

**Fig 2:**
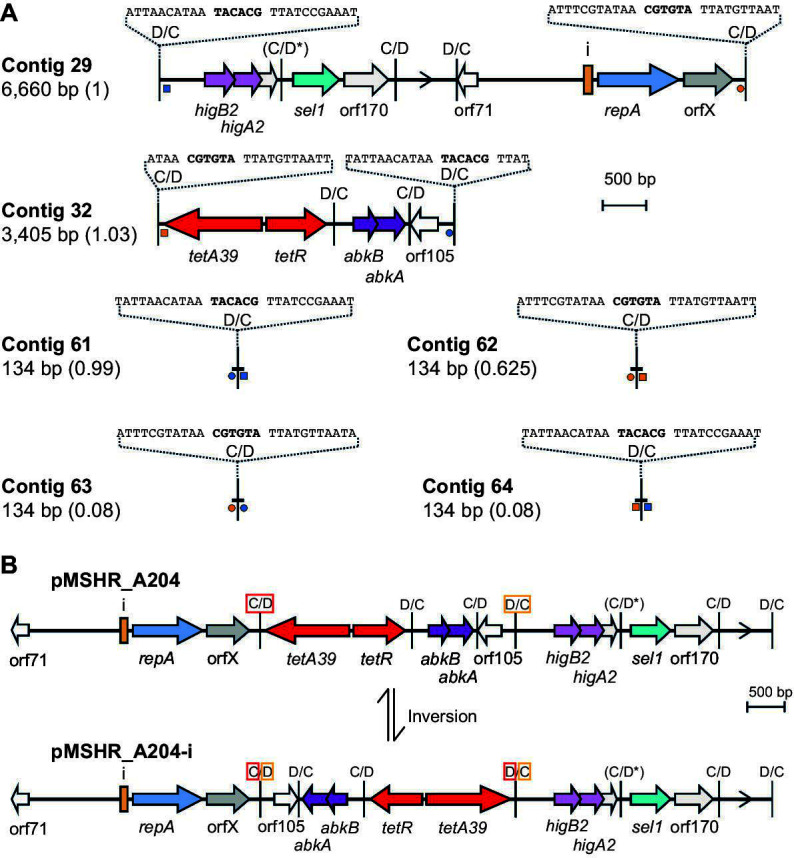
Two potential configurations of pMSHR_A204. (**A**) Contigs containing p*dif* sites in MSHR_A204. (**B**) Structure of the two potential configurations of the plasmid, pMSHR_A204 (top) and pMSHR_A204-i (bottom). Arrows indicate the extent and orientation of genes and open reading frames with their names below. The *repA* gene is blue, antibiotic resistance genes are red, putative toxin-antitoxin genes are purple, *sel1* is teal, orfX is gray, and open reading frames that encode a protein of unknown function are white and are numbered according to the size (aa) of their encoded protein. The iteron region is indicated by an orange box with an i above. An open arrow represents the relative orientation for the *dif* module that contains no reading frames. The p*dif* sites are indicated by a vertical bar with the orientation of the site (C/D or D/C) indicated above. (C/D*) indicates that a conserved base in the XerD binding site is mutated and the site may not be functional. In panel **A**, the blue and orange squares or circles indicate contig ends that are the same. The sequences of relevant p*dif* sites are indicated above. In panel **B**, the D/C and C/D p*dif* sites surrounding the region potentially inverted in pMSHR_A204 are indicated by red and orange boxes, respectively.

The *A. baumannii* strain MSHR_A204 carries the *tet39* tetracycline resistance determinant, and no other acquired antibiotic resistance genes were identified in the draft genome (28). Here, the *tet39* determinant was found to be located on a 3,405 bp contig that did not include a *repA* gene (Fig. 2A). The *Acinetobacter* plasmid typing tool detected a single contig of 6,660 bp containing a *repA* gene (Fig. 2A) in the MSHR_A204 genome. This *repA* was identical to the *repA* of the r3-T5 type reference plasmid pABLAC1 (CP007713). Both the *tet39* and *repA* contigs had a coverage ~6-fold higher than that of the chromosome, indicating a plasmid copy number of approximately 6. Both ends of each contig include most of a p*dif* site (Fig. 2A), and four additional small contigs (134 bp) that each included a p*dif* site with a small amount of sequence on either side were found in the draft genome (Fig. 2A). Analysis of these small contigs revealed that they each included one end of the *repA* contig and one end of the *tet39* contig (the four ends are indicated by different symbols in Fig. 2A), indicating that the plasmid could potentially be assembled in two ways as shown in Fig. 2B. To determine the relative abundance of these two configurations in the genome, the read depth for the small contigs was used. Contig 61 and contig 62 have a coverage of 0.99 and 0.625, respectively, relative to the *repA* contig, whereas the coverage of contigs 63 and 53 was only 0.08-fold. Hence, both configurations were present in the DNA sequenced, but the second configuration was present in only ~8% of cases. The two possible configurations differ by the inversion of a 3.4 kbp segment bounded by two of the six p*dif* sites identified in pMSHR_A204 (boxed in Fig. 2B). Therefore, the plasmid was assembled using the majority form predicted by the genome data and submitted to GenBank under accession number PP526176.

**Fig 3:**
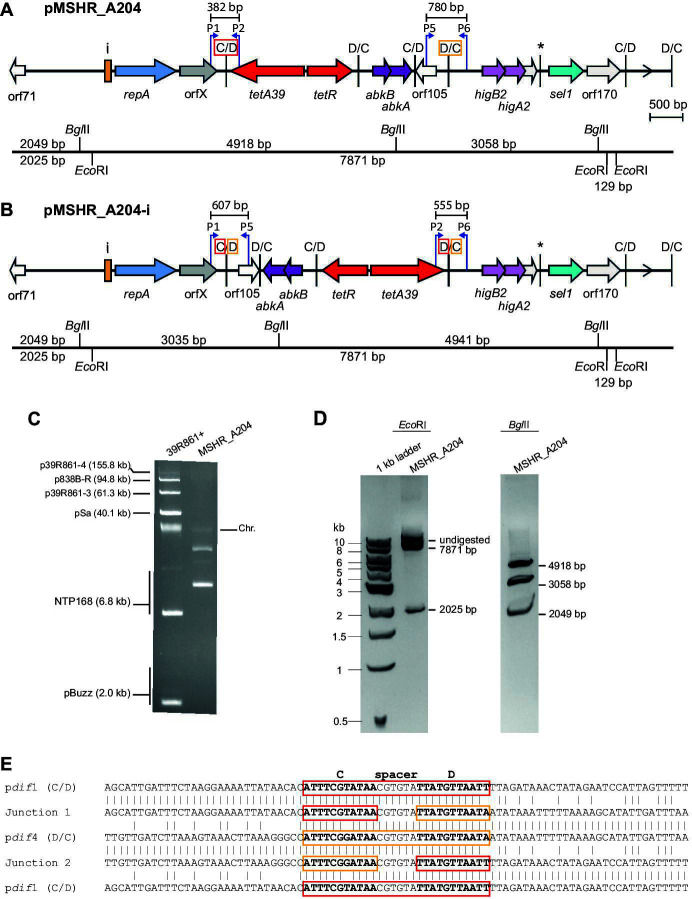
Plasmid DNA from MSHR_A204. Structure of pMSHR_A204 and location of restriction sites in (**A**) configuration 1 and (**B**) configuration 2 of pMSHR_A204. Arrows indicate the extent and orientation of genes and open reading frames with their names below and are colored as in Fig. 2. The iterons, p*dif* sites, and the orientation of the empty *dif* module are indicated as in Fig. 2. The D/C and C/D sites surrounding the region potentially inverted in pMSHR_A204 are indicated by red and orange boxes, respectively. The * above a p*dif* site indicates that the site may not be functional due to mutation of a conserved base in the XerD binding site. Bent arrows indicate the location and direction of primers used to amplify across p*dif* sites, with the primers labeled as P1, P2, P5, and P6. The PCRs used to determine if inversion occurred between p*dif* site 1 and p*dif* site 4 are indicated by a horizontal bar with the expected product size above. The location of *Bgl*II and *Eco*RI restriction sites is indicated below. The fragment sizes generated by restriction digestion of plasmid DNA with either *Bgl*II or *Eco*RI are indicated above or below the line, respectively. pMSHR_A204 is drawn to scale from GenBank accession number PP526176. Gel electrophoresis of (**C**) uncut and (**D**) *Bgl*II-digested (right) or *Eco*RI-digested (left) pMSHR_A204 plasmid DNA. Plasmid DNA extracted from 39R861+ was used as a plasmid size standard, and NEB 1 kb ladder was used as a linear size marker. The band sizes of the standards are indicated. (**E**) Sequences of PCR products showing recombination at the XerC/XerD binding sites. Sequences of p*dif* site 1 and p*dif* site 4 and ~25 bp on either side are aligned with the sequences of DNA from PCR products P1–P5 (junction 1) and P2– P6 (junction 2). The p*dif*4 and junction 2 sequences are inverted to enable comparison. In the sites from configuration 1 (pdi*f1* and p*dif*4), the XerC and XerD binding sites are surrounded by red (p*dif*1) or orange (p*dif*4) boxes with C, spacer, and D indicated above. The red/orange boxes around the XerC and XerD binding sites in junction 1 and junction 2 indicate the origin (p*dif*1 and p*dif*4) of that part of the site. Bases shared are indicated by vertical lines between two sequences.

The presence of a single plasmid of the expected size of 10 kbp was confirmed by visualization after electrophoretic separation of plasmid DNA extracted from MSHR_A204 (Fig. 3C). Digestion with EcoRI or BglII, which each cut three times in pMSHR_A204, yielded bands of the predicted sizes (Fig. 3D; for expected sizes see Fig. 3A and B). However, digestion with these enzymes could not distinguish between the expected majority and minority configurations due to the similarity of the fragment sizes of the BglII digests of the two, which would be indistinguishable by gel electrophoresis.

### pMSHR_A204 is an r3-T5 plasmid with five accessory *dif* modules

pMSHR_A204 is 10,025 bp in length. The backbone is 3,267 bp (including 1 of the 28 bp p*dif* sites) and is a C module. It is an r3-T5 type plasmid with just one nucleotide difference and one base missing relative to the r3-T5 reference plasmid pABLAC1 backbone sequence. Hence, as described recently for pABLAC1 (4), the pMSHR_A204 backbone includes four copies of the 22 bp iteron sequence (5′- TAAAACGAGGTTTACCTTGCAT-3′) located 56 bp upstream of *repA* and has the orfX open reading frame downstream of *repA*. These features make it a member of the Rep_3/OrfX group as defined recently (4). An open reading frame encoding a 71 aa protein of unknown function is located at the other end of the backbone (Fig. 2 and 3).

The rest of pMSHR_A204 consists of an accessory region comprised of five *dif* modules, three D modules (including the *tet39* module), and two C modules (Fig. 2B). The largest C module, which contains *higBA2-*orf69-*sel1*-orf170, is known to be made up of three *dif* modules (two C modules and one D module) that have been fused together by a deleterious mutation in one p*dif* site (the C/D* site in Fig. 2; indicated by * in later figures) and the deletion of another p*dif* site (between *sel1* and orf170) (3). A second toxin-antitoxin gene pair, *abkBA*, present in pMSHR_A204 is also found in a C module that is only 90%–95% identical to *abkBA* modules detected previously (3). The remaining modules are a D module that carries an open reading frame (orf105) encoding a hypothetical protein of 105 aa and a D module that does not contain any predicted open reading frames. The orientation of the latter is indicated by an arrowhead in the figures.

**Fig 4:**
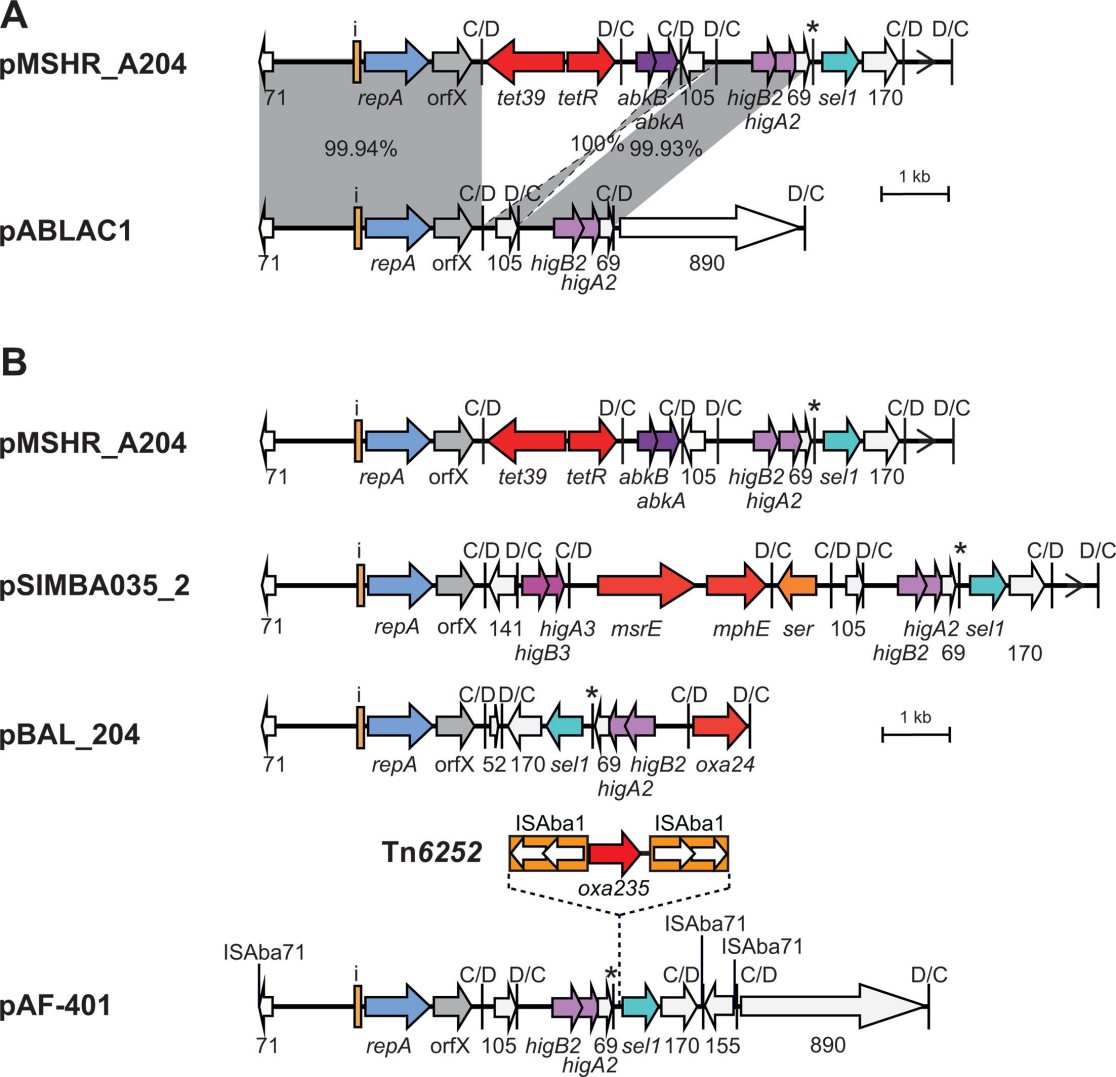
r3-T5 plasmids carrying antibiotic resistance genes. (**A**) Comparison of pMSHR_A204 and pABLAC1. (**B**) Structure of r3-T5 plasmids with antibiotic resistance genes. The extent and orientation of genes and open reading frames are indicated by arrows with their names below. *repA*, orfX, antibiotic resistance determinants, toxin-antitoxin gene pairs, *sel1*, and open reading frames of unknown function are colored as in Fig. 2. The *ser* gene, encoding a serine recombinase, is orange. The iterons, p*dif* sites, and the orientation of the empty *dif* module are indicated as in Fig. 2. The * above a p*dif* site indicates that the site may not be functional due to mutation of a conserved base in the XerD binding site. Insertion sequence ISAba1 is indicated by an orange box with the transposase gene indicated by white arrows within the box. The location of other insertion sequences is indicated by a vertical bar with the name above. In panel **A**, regions with significant DNA identity are indicated by gray shading, with the DNA identity (%) indicated. Drawn to scale from GenBank accession numbers listed in Table 2.

The overall structure and composition of pMSHR_A204 was compared to that of pABLAC1, which is the exemplar for the r3-T5 group. pABLAC1 is from the ST10:KL49:OCL2 *A. baumannii* isolate LAC-4, but p*dif* sites were not previously reported (29). pABLAC1 includes one C module and two D modules (Fig. 4A). Like pMSHR_A204, pABLAC1 carries *higBA2*-orf69 but in the original C module which is bounded by two complete p*dif* sites. The orf105 module is also shared, but it is in opposite orientations relative to the backbone module (Fig. 4A). The second D module of pABLAC1 carries a large orf of unknown function.

### Rearrangements of the pMSHR_A204 configuration

We attempted to recover the two forms of pMSHR_A204 detected in the sequence data by transformation of MSHR_A204 plasmid DNA into the tetracycline-susceptible *A. baumannii* strain AB307-0294. However, using electroporation, the transformation efficiency was very low, with only a total of 10 Tc^R^ transformants obtained from three transformation reactions (1 μg/ reaction). In contrast, the 6,078 bp pRAY* (30) used as a control had a high transformation efficiency, with 1 × 10^4^–9× 10^5^ Km^R^ transformants/μg of DNA under the same conditions. Gel electrophoresis and restriction digestion of plasmid DNA obtained from the transformants produced the same results as for plasmid DNA from the original strain (data not shown). However, while a single transformant would take up a single plasmid molecule, PCR amplification across the p*dif* site junctions in both the original strain and transformant (see Fig. 3A and B for the location of primers and predicted amplicon sizes) produced a band of the expected size for both configurations of the plasmid shown in Fig. 2B and 3, indicating that both forms of the plasmid are present shortly after transformation. Sequencing of the PCR products from both the original strain and the transformants indicated that the recombination crossover occurred within the p*dif* sites which have identical 6 bp spacers (Fig. 3E).

**Fig 5:**
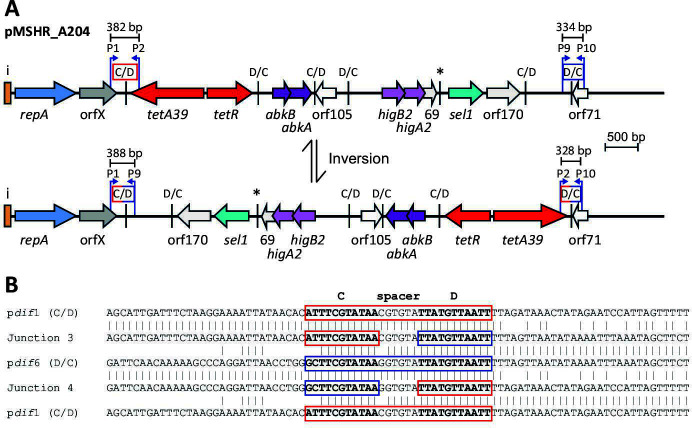
Inversion between p*dif* site 1 and p*dif* site 6 in pMSHR_A204. (**A**) Structure of pMSHR_A204 (top) and the predicted structure after recombination between p*dif* site 1 and p*dif* site 6 (bottom). The extent and orientation of genes and open reading frames are indicated by colored arrows, as in Fig. 2 and 3. Bent arrows indicate the location and direction of primers used to amplify across p*dif* sites with the primers P1, P2, P9, and P10. The PCRs used to determine whether an inversion event had occurred are indicated by a horizontal bar with the product size above. (**B**) Sequences of PCR products showing recombination at p*dif* site 1 and p*dif* site 6. Sequences of p*dif* site 1 and p*dif* site 6 and ~25 bp on either side are aligned with the sequences of DNA from the PCR products P1–P9 (junction 3) and P2–P10 (junction 4). In the original sites, the extent of the p*dif* sites is indicated by red (p*dif*1) or blue boxes (p*dif*6) with C, spacer, and D indicated above. The orange/blue boxes around the XerC and XerD binding sites in junctions 3 and 4 indicate the origin (p*dif*1 or p*dif*6) of that part of the site. Bases shared are indicated by vertical lines between two sequences. p*dif* site 6 and junction 4 sequences are inverted to enable comparison, and so the sites are in the XerC binding site–spacer–XerD binding site orientation. The * above a *pdif* site indicates that the site may not be functional due to mutation of a conserved base in the XerD binding site.

The sequences of the six p*dif* sites in pMSHR_A204 are shown in Table 1. As the spacers in the inversely oriented sites 1 (C/D) and 6 (D/C) had nearly identical spacers (five of six residues match), we tested whether an inversion generated by recombination between them, which would invert the orientation of the backbone relative to the complete *dif* module set, could be detected. The inversion configuration and the primers and amplicon sizes are indicated in Fig. 5A. Amplicons of the predicted sizes were detected, and the sequences of the junctions found in these amplicons (compared in Fig. 5B) show that inversion occurred with a crossover within the p*dif* sites. The single mismatch in the spacers adjacent to the XerC binding site did not preclude recombination between the two sites.

**TABLE 1:** p*dif* sites in pMSHR_A204

p*dif* site	Orientation	XerC binding site	Spacer	XerD binding site
1	C/D	ATTTCGTATAA	CGTGTA	TTATGTTAATT
2	D/C	GTTTCGTATAA	GCGCTA	TTATGTTAAAT
3	C/D	GATTCGTATAA	CGCACA	TTATGTTAAAT
4	D/C	ATTTCGGATAA	CGTGTA	TTATGTTAATA
5	C/D	ATTTCATATAA	CGCACA	TTATGTTAAAT
6	D/C	GCTTCGTATAA	GGTGTA	TTATGTTAATT
*^a^	C/D*	GATTCGCATAA	GAGTTT	CTATGTTAAAT

^*a*^ This site is likely inactive due to mutation of a conserved base in the XerD binding site. The base is underlined in the sequence.

**Fig 6:**
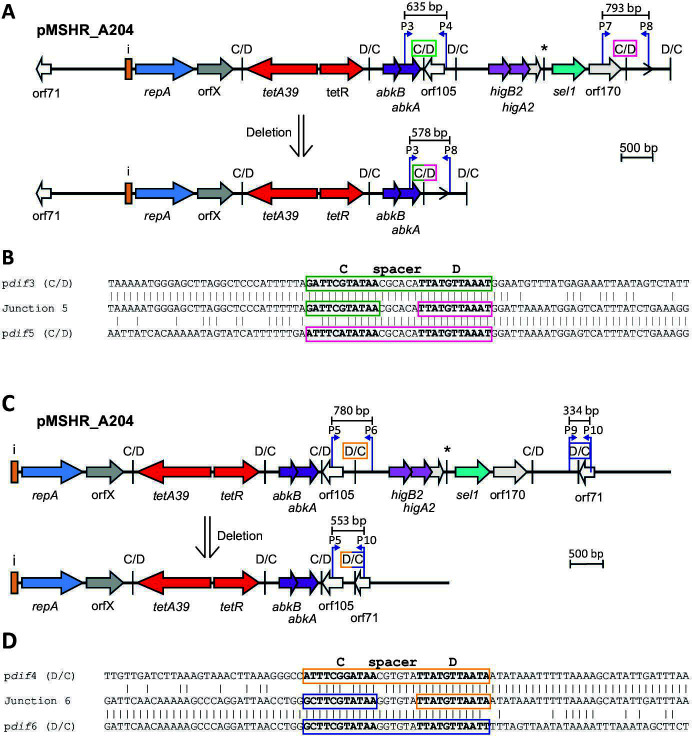
Excision of *dif* modules from pMSHR_A204. (**A**) Excision of two modules by recombination between p*dif*3 and p*dif*5. Structure of pMSHR_A204 (top) and the predicted structure after recombination between p*dif* sites 3 and 5 (bottom). The extent and orientation of genes and open reading frames are indicated by colored arrows and p*dif* sites are indicated as in Fig. 2 and 3. Bent arrows indicate the location and direction of primers used to amplify across p*dif* sites with the primers labeled as P3, P4, P7, and P8. The PCRs used to determine if excision occurred between p*dif* site 3 and p*dif* site 5 are indicated by a horizontal bar with the expected product size above. (**B**) Sequences of PCR products showing recombination at the p*dif*3 and p*dif*5 XerC/XerD binding sites. Sequences of p*dif* site 3 and p*dif* site 5 and ~25 bp on either side are aligned with the sequence of DNA from the PCR product P3–P8 (junction 5). The extent of the original p*dif* sites is indicated by green (p*dif*3) or pink boxes (p*dif*5) with C, spacer and D indicated above. The green/pink boxes around the XerC and XerD binding sites in junction 5 indicate the origin (p*dif*3 or p*dif*5) of that part of the site. Bases shared are indicated by vertical lines between two sequences. (**C**) Excision of two modules by recombination between p*dif* site 4 and p*dif* site 6. Structure of pMSHR_A204 (top) and the predicted structure after recombination between p*dif* sites 4 and 6 (bottom). Bent arrows indicate the location and direction of primers used across p*dif* sites with primers P5, P6, P9, and P10. (**D**) Sequences of PCR products showing recombination between p*dif* site 4 and p*dif* site 6. Sequences of p*dif* site 4 and p*dif* site 6 and surrounds are aligned with the sequence of the PCR product P5–P10 (junction 6). The extent of the original p*dif* sites is indicated by an orange (p*dif*4) or blue (p*dif*6) box. The orange/blue boxes around the XerC and XerD binding sites in junction 6 indicate the origin (p*dif*4 or p*dif*6) of that part of the site. Bases shared are indicated by vertical lines between two sequences. All three sequences are inverted to enable comparison, and so the sites are in XerC binding site–spacer–XerD binding site orientation. The * above a *pdif* site indicates that the site may not be functional due to mutation of a conserved base in the XerD binding site.

We have previously shown that two adjacent *dif* modules (one C module plus one D module) can be excised (6). Hence, we also examined loss of the adjacent module pairs flanked by the p*dif* sites 3 and 5 or 4 and 6, as these sites are in direct orientation and include spacers that are identical or differ at a single position. Using primers shown in Fig. 6A and C, amplicons of the predicted sizes were obtained and comparison of the junctions in them revealed that in each case the crossover was centered on the p*dif* sites (Fig. 6B and D). This provides further evidence that *dif* modules can be excised when the requirement for direct orientation between the sites and high identity in the spacer is satisfied. The requirement for directly oriented p*dif* sites necessitates that two standard modules (one C plus one D) are excised together, as predicted previously (5).

### r3-T5 plasmids are widespread

To further examine the diversity of plasmid backbones and *dif* module carriage of plasmids in the r3-T5 group, all plasmids with a *repA* gene with >95% DNA identity to the *repA* sequence of pABLAC1 were downloaded from the GenBank non-redundant nucleotide database (last searched 30 April 2025). A total of 43 additional plasmids were found, bringing the total number of plasmids in the group to 45 (Table 2). These plasmids were from *A. baumannii* (*n* = 33), *A. pittii* (*n* = 10), *A. seifertii* (*n* = 1) strains, and an undetermined *Acinetobacter* species (*n* = 1). The strains were isolated between 1997 and 2022 from both clinical and environmental sources and were from 27 countries and all inhabited continents: Australia, Africa (Tanzania), Asia (China, India, Singapore, South Korea, Taiwan, Vietnam), Europe (Czech Republic, France, Finland, Germany), North and Central America (Honduras, Mexico, and USA) and South America (Brazil, Chile). The plasmid pD1279779, like pMSHR_A204, was recovered from an *A. baumannii* infection that was acquired in the community in the Northern Territory of Australia (31).

**TABLE 2:** r3-T5 plasmid sequences in GenBank^*m*^

Plasmid	Size (bp)	Species	Year of isolation	Country	Isolation source^*a*^	Accession
pABLAC1	8,006	*A. baumannii*	1997	USA	Human	CP007713
pMSHR_A204	10,025	*A. baumannii*	2011	Australia	Blood	PP526176
pBAL_204	7,155	*A. baumannii*	2010	Vietnam	VAP BAL^*a*^	KT946773
pSIMBA035_2	12,185	*A. baumannii*	2008	Singapore	Human	CP161997
pAF-401	17,583	*A. baumannii*	2009	Mexico	Small colon	CP018255
pAba10324b	7,143	*A. baumannii*	2012	Mexico	Bronchial fluid	CP023024
pJBAB22^*b*^	7,146	*A. baumannii*	2022	South Korea	Plant	CP138325
pDETAB6^*b*^	7,145	*A. baumannii*	2019	China	–^*l*^	CP072529
plas2^*c*^	7,146	*A. baumannii*	2021	China	Soil	CP150099
pPUMA0247_1^*c*^	7,146	*A. baumannii*	2019	–	–	CP144908
p4-G6290164	8,633	*A. baumannii*	2015	Brazil	Rectal swab	CP167774
BHS4 p1^*d*^	7,145	*Acinetobacter sp*.	2020	China	AC condensate^*a*^	CP075324
pAP43-3^*e*^	9,203	*A. pittii*	2018	China	Urine	CP043055
pWZ-02^*e*^	9,202	*A. pittii*	2015	China	Human	CP168072
p2_005069	9,203	*A. pittii*	–	China	Human	CP026088
p2014S07-126-4^*f*^	9,203	*A. pittii*	2014	Taiwan	Urine	CP131877
p1DB008	9,203	*A. baumannii*	–	Germany	–	CP087342
pD1279779	7,416	*A. baumannii*	–	Australia	Human	CP003968
pPM194229_3	9,847	*A. baumannii*	2019	India	BAL^*a*^	CP050435
pApiMCR8900a	7,614	*A. pittii*	2015	Honduras	Wound	CP139250
pY03-03	8,638	*A. baumannii*	2021	China	Chicken	CP163385
p3LMG994	7,296	*A. baumannii*	–	Germany	–	CP087334
pSIMBA003_2^*g*^	7,596	*A. baumannii*	2006	Singapore	Human	CP161988
pXH2146-2^*g*^	7,596	*A. baumannii*	2021	China	Human	CP128374
pAB17961-2	6,667	*A. baumannii*	2016	USA	Blood	CP065434
pApiAN37c	5,061	*A. pittii*	2014	Mexico	Peritoneal dialysis fluid	CP139279
p4UC20804	6,829	*A. baumannii*	2010	Chile	Peritoneal abscess	CP076811
p2OC074	6,806	*A. baumannii*	–	Germany	–	CP087330
pB8300	25,150	*A. baumannii*	2015	India	Blood	CP021348
p2_100004	18,485	*A. pittii*	2015	China	Human	CP027248
pEH_gr3	25,856	*A. baumannii*	2018	Czech Republic	Human	CP038260
pAS6-4	20,374	*A. seifertii*	2010/17^*h*^	Taiwan	Blood	CP061695
pKCRI-28-1	29,606	*A. baumannii*	2013	Tanzania	Wound swab	LR026972
F-1629-p^*d*^	19,011	*A. baumannii*	–	South Korea	Human	CP099970
AB105-p4^*d*^	13,439	*A. baumannii*	2022	China	Human	CP103341
13A297n-p1^*i*^	16,101	*A. baumannii*	2013	France	Urine	CP129247
p6E072658	18,806	*A. baumannii*	–	Finland	Pulp paper mill	CP061708
plas3_LRB^*j*^	19,102	*A. baumannii*	2020	China	Wastewater	CP121373
plas3_Ab_8_4^*j*^	19,102	*A. baumannii*	2020	China	Wastewater	CP121368
pOXA58_005069	112,436	*A. pittii*	–	China	–	CP026086
pCARB128_4^*k*^	17,758	*A. pittii*	2020	Germany	Human	CP139943
pSP304-3^*d*^^,*k*^	9,185	*A. baumannii*	2019	India	Sputum	CP040083
pSP304-2^*d*^^,*k*^	9,605	*A. baumannii*	2019	India	Sputum	CP040082
pSP304-1^*d*^^,*k*^	11,933	*A. baumannii*	2019	India	Sputum	CP040081
p2014S06-099-3^*k*^	7,849	*A. pittii*	2014	Taiwan	Human	CP033543

^*a*^ VAP, ventilator-associated pneumonia; BAL, bronchoalveolar lavage; AC, air conditioner.

^*b*^ The pJBAB22 and pDETAB6 sequences are almost identical (six SNDS and one base indel, 99.90% DNA identity) across their full length.

^*c*^ The pPUMA0247_1 and plas2 sequences are almost identical (three SNDs, 99.96% DNA identity) across their full length.

^*d*^ Plasmid is unnamed in GenBank, named here.

^*e*^ The pAP43-3 and pWZ-02 sequences are almost identical (one SND and one base indel, 99.98% DNA identity) across their full length.

^*f*^ Two p2014S07-126-4 sequences are available in the GenBank non-redundant database, a second less accurate sequence is available under accession number CP033534.

^*g*^ The pXH2146-2 and pSIMBA003_2 sequences are identical across their full length.

^*h*^ Isolated between 2010 and 2017, exact year of isolation is unknown.

^*i*^ Plasmid is unnamed in GenBank, named in (3).

^*j*^ The plas3_LRB and plas3_Ab_8_4 sequences are identical across their full length.

^*k*^ The plasmid sequence was excluded from further analysis due to sequence errors, poor quality sequence, or assembly issues.

^*l*^ –, no information available.

^*m*^ Gray shade indicates the cointegrates.

### General characteristics of r3-T5 plasmids

The 45 r3-T5 plasmids range in size from 5,061 bp to 112,436 bp (Table 2). Five plasmid sequences were excluded from further analysis due to likely sequencing or assembly issues (see Materials and Methods for details), and five pairs of plasmids were identical or nearly identical (>99.9% DNA identity; indicated in Table 2), reducing the number of unique plasmid configurations examined to 35. Screening for additional *repA* genes revealed that a second Rep_3 type replication protein was encoded by 11 of the longer r3-T5 plasmid types, indicating that they were cointegrates. Thus, cointegrates that include a second backbone are quite common, with 31% (11/35) of the R3-T5 type plasmids having two *repA* genes. The longest of these (pOXA-58_005069, 112 kbp) contains an R3-T28 type plasmid but was not examined further. The remaining 10 types are described in more detail below.

The 24 plasmid configurations with only a single *repA* gene (top group in Table 2) ranged in size from 5,061 bp to 17,583 bp; only two were larger than the 10,025 bp pMSHR_A204. Other features of plasmids in this group are summarized in Table 3. The backbones of most of them are of very similar length (3,266 bp–3,270 bp) and exhibit high levels of identity, generally sharing >99% identity with the backbone of the pABLAC1 reference (Table 3). The lower values reflect the presence of one or more short recombination patches (Fig. S1) rather than divergence along the full length.

**TABLE 3:** Additional information about the r3-T5 non-cointegrate plasmids

Plasmid	*repA* identity (%)^*a*^	No of iterons	R3-T5 backbone		No. of *dif* modules^*c*^	Antibiotic resistance genes in *dif* modules	Toxin/antitoxin module
			Size (bp)^*b*^	Identity (%)^*a*^			
pABLAC1	100	4	3,268	100	3	–^*f*^	*higBA2*
pMSHR_A204	100	4	3,267	99.94	5	*tet39*	*higBA2, abkBA*
pBAL_204	99.78	4	3,268	99.57	3	*oxa24*	*higBA2*
pSIMBA035_2	100	4	3,268	99.97	7	*msrE-mphE*	*higBA2, higBA3*
pAF-401	100	4	3,267^*d*^	99.97	4	*oxa235^e^*	*higBA2*
pAba10324b	100	4	3,268	99.97	3	–	*higBA2*
pJBAB22	100	4	3,268	99.97	3	–	*higBA2*
pDETAB6	100	4	3,268	99.88	3	–	*higBA2*
plas2	99.89	4	3,268	99.97	3	–	*higBA2*
pPUMA0247_1	99.89	4	3,268	99.94	3	–	*higBA2*
p4_G6290164	99.89	4	3,268	99.94	3	–	*higBA2*
pBHS4-1	99.89	4	3,267	99.85	3	–	*higBA2*
pAP43-3	100	4	3,268	99.97	3	–	*higBA2*
pWZ-02	99.89	4	3,268	99.94	3	–	*higBA2*
p2_005069	100	4	3,268	99.97	3	–	*higBA2*
p2014S07-126-4	100	4	3,268	99.97	3	–	*higBA2*
p1DB008	99.78	4	3,268	99.85	3	–	*higBA2*
pD1279779	100	4	3,268	99.97	3	–	*higBA2*
pPM194229_3	100	4	3,268	100	4	–	*higBA2*
pApiMCR8900a	99.89	4	3,267	99.63	4	–	*higBA2*
pY03-03	99.89	4	3,268	99.78	4	–	–
p3LMG994	99.89	4	3,270	98.15	3	–	*higBA5*
pSIMBA003_2	99.78	4	3,267	99.72	4	–	*higBA2*
pXH2146-2	99.78	4	3,267	99.72	4	–	*higBA2*
pAB17961-2	99.78	4	3,266	99.51	2	–	*higBA5*
pApiAN37c	99.68	4	3,267	99.41	2	–	*higBA3*
p4UC20804	99.68	4	3,266	99.35	2	–	–
p2OC074	99.57	4	3,267	98.39	2	–	*abkBA*

^*a*^ Nucleotide identity compared to the representative r3-T5 plasmid pABLAC1 (CP007713).

^*b*^ Backbone size includes only one of the adjacent 28 bp p*dif* sites.

^*c*^ The r3-T5 backbone module was not included in the number of *dif* modules. The *higBA2-sel1*-orf170 fusion module described previously (3) was counted as a single module.

^*d*^ ISApi2 interrupts the backbone; to calculate the backbone size ISApi2 (1,482 bp) and one copy of the 5 bp TSD were removed. ISApi2 targets the C side of the p*dif* site (32, 33).

^*e*^ *oxa235* is in Tn*6252,* which has inserted into the *higBA2-sel1*-orf170 fusion module 98 bp upstream of *sel1*.

^*f*^ –, no information available.

The 24 non-cointegrate plasmid sequences were each annotated, and p*dif* sites were identified manually. Most plasmids carry an odd number of accessory *dif* modules as expected, granted that C and D modules must alternate to generate complete p*dif* sites; 13 types carry three *dif* modules, only pMSHR_A204 carries five, and pSIMBA035_2 carries seven. The remaining 9 types carry two (*n* = 4) or four (*n* = 5) *dif* modules due to the presence of a CD fusion module, as shown in Fig. 1B. All but 2 carry at least one *dif* module that includes a toxin-antitoxin gene pair (Table 3), and for most, this is the *higBA2* pair.

### Antibiotic resistance genes in r3-T5 plasmids

A few of the non-cointegrate plasmids, all derived from an *A. baumannii* from a human source (see Table 2), included an antibiotic resistance gene (Table 3), and these are compared to pMSHR_A204, which carries the *tet39* module, in Fig. 4B. pSIMBA035_2 (12,185 bp) was isolated in Singapore and includes seven *dif* modules (three C modules and four D modules), three of which are present in pMSHR_A204, although the orf105 module is inverted. Two of the *dif* modules (*tet39* and *abkBA*) in pMSHR_A204 have been replaced by four *dif* modules, one of which includes the *msrE-mphE* genes that confer resistance to macrolides, and another represents a second toxin-antitoxin module (*higBA3*).

Plasmid pBAL_204 (7,155 bp), recovered in Vietnam from a clinical *A. baumannii* isolate BAL_204 (ST755:KL55:OCL6) (34), carries the D module that includes the *oxa24* gene conferring resistance to carbapenem antibiotics (7). The *higBA2-*orf69-*sel1*-orf170 fusion module is the only module shared by pMSHR_A204, pSIMBA035_2, and pBAL_204. The third module in pBAL_204 includes a short orf (Fig. 4B).

In the final large plasmid pAF-401 (17,583 bp), the resistance gene is not naturally part of the *dif* module. A known transposon, Tn*6252*, that includes the *oxa235* carbapenem resistance gene surrounded by inversely oriented copies of ISAba1 (29, 35) is inserted between orf69 and *sel1* in the large *higBA2-*orf69-*sel1*-orf170 fusion module, creating a duplication of the 9 bp target (TCTAATTTT). This plasmid also includes three copies of ISAba71, first identified in plasmid pS30-1 (5), which is a member of a group of insertion sequences that target both *dif* and p*dif* sites, inserting 5 bp from the XerC binding site (32). With these additions removed, the progenitor plasmid would be only 9,837 bp, with four accessory *dif* modules, including the large *higBA2* fusion module and the orf105 module found in pMSHR_A204, as well as a small CD module explaining the presence of an even number of accessory modules.

### *dif* module configurations in non-cointegrate r3-T5 plasmids

**Fig 7:**
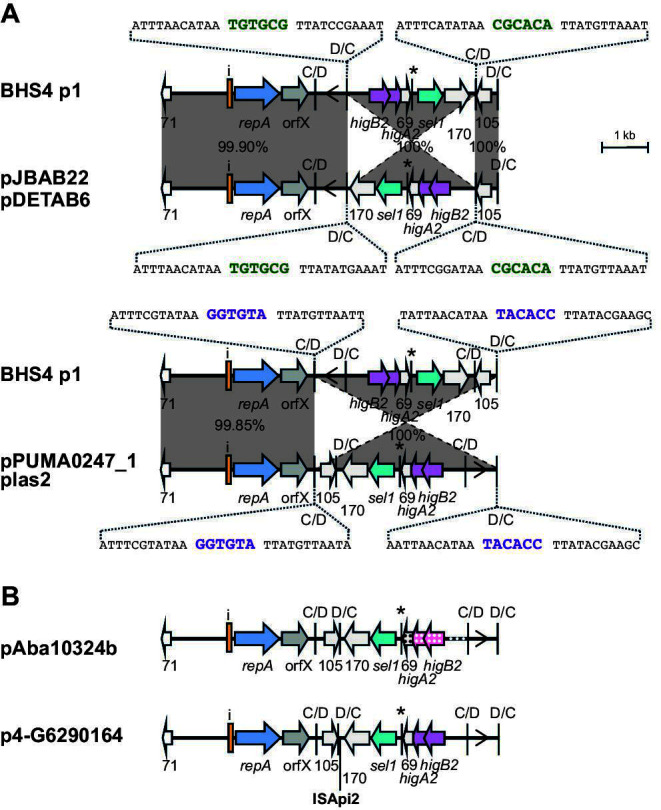
*dif* module rearrangements in r3-T5 plasmids with the same set of *dif* modules. (**A**) Inversion of one (top) or three *dif* modules (bottom). (**B**) Structure of r3-T5 plasmids with a diverged region (pAba10324b) or additional insertion (p4-G6290164) compared to plasmids in panel **A**. The extent and orientation of genes and open reading frames are indicated by arrows with their names below and colored as in Fig. 2. The iterons, p*dif* sites, and the orientation of the empty *dif* module are indicated as in Fig. 2. The * above a p*dif* site indicates that it may not be functional due to mutation of a conserved base in the XerD binding site. The location of ISApi2 in p4-G6290164 is indicated by a vertical bar with the name below. In panel **A**, regions of significant DNA identity are indicated by gray shading, with the % identity indicated. The sequence of the p*dif* sites involved in the inversion or replacement events is shown, spacers that have the same sequence are purple or green. In panel **B**, the region where the *dif* module is diverged is indicated by a polka dot pattern. Drawn to scale from GenBank accession numbers listed in Table 2.

**Fig 8:**
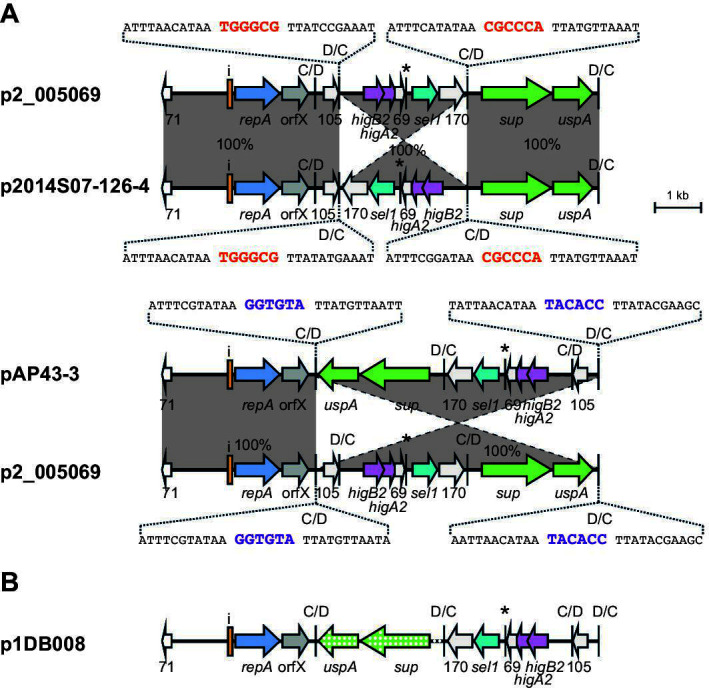
*dif* module rearrangements in r3-T5 plasmids that carry the *sup-uspA* module. (**A**) Inversion of one (top) or three (bottom) *dif* modules in plasmids with the same *dif* module content. (**B**) Structure of p1DB008, which has a diverged *sup-uspA* module. The extent and orientation of genes and open reading frames are indicated by arrows with their names below and colored as in Fig. 2. The *sup* (encoding a sulfate permease) and *uspA* genes are light green. Iterons and p*dif* sites are indicated as in Fig. 2. The * above a p*dif* site indicates that it may not be functional due to mutation of a conserved base in the XerD binding site. In panel **A**, regions of significant DNA conservation are indicated by gray shading, with the DNA identity (%) indicated. The sequence of the p*dif* sites involved in each rearrangement event is shown, spacers that have the same sequence are indicated in purple or orange. In panel **B**, the region where the *dif* module is diverged is indicated by a polka dot pattern. Drawn to scale from GenBank accession numbers listed in Table 2.

The structures of each of the remaining 19 unique plasmid types were compared. A few sets of plasmids were found to carry the same *dif* module complement, which is either arranged in a different configuration or includes one module with a significantly diverged sequence. In these sets, where inversions have arisen, examination of the p*dif* sequences revealed that they arose via recombination between pairs of inversely oriented p*dif* sites that have identical spacer sequences. Examples shown involved inversion of either one *dif* module or three *dif* modules (Fig. 7A and 8A). Further related plasmids (Fig. 7B and 8B) include recombination patches that have introduced a diverged segment, or an insertion sequence has been acquired.

**Fig 9:**
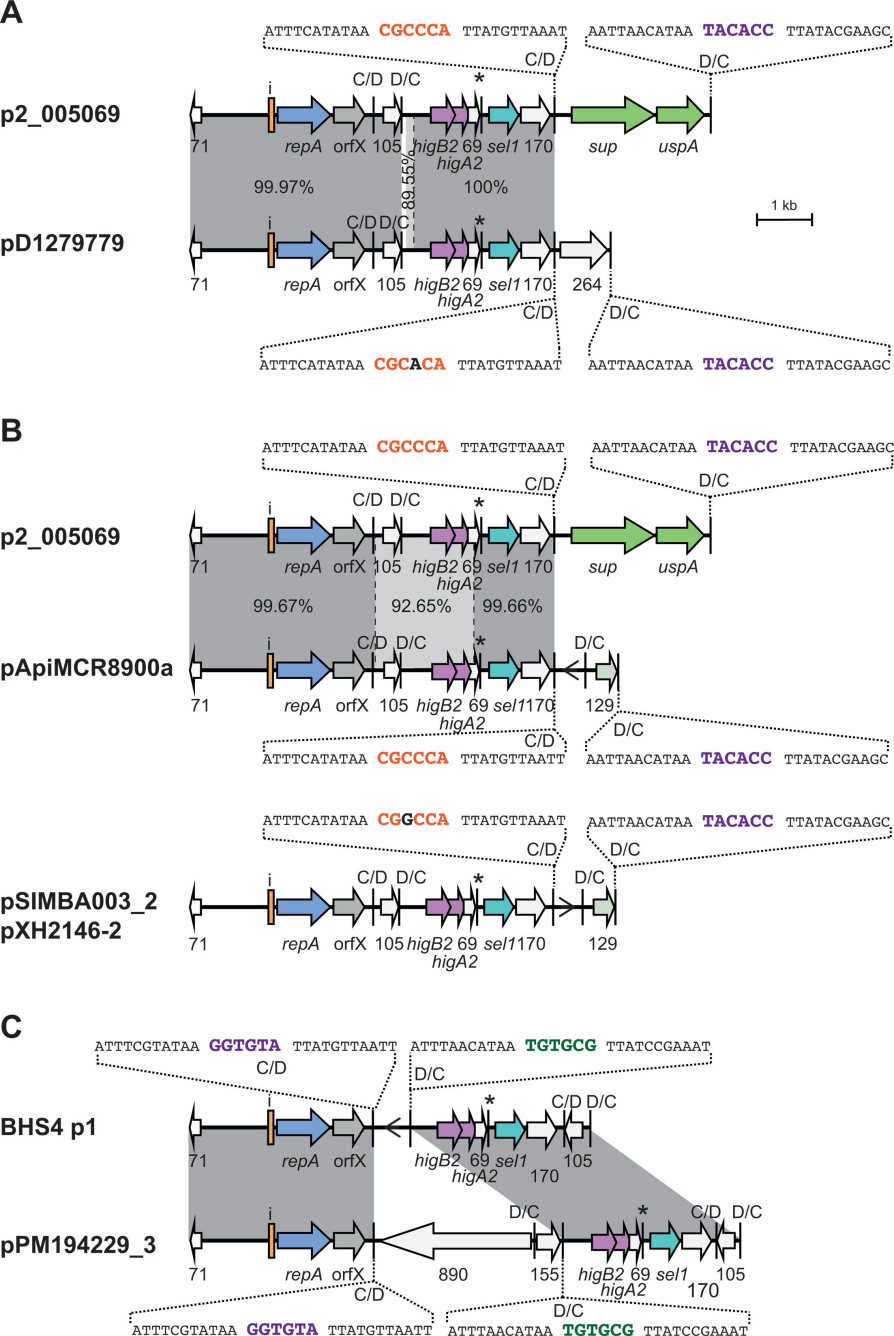
Replacement of dif modules between closely related plasmids. (**A**) Replacement of a single *dif* module in p2_005069 with (**A**) a single module in pD1279779 and (**B**) two modules (one C and one CD) in pApiMCR8900a. (**C**) Replacement of a single *dif* module between BHS4 p1 and pPM194229_3. The extent and orientation of genes and open reading frames are indicated by arrows with their names below and are colored as in Fig. 2 and 8. The iterons, p*dif* sites, and the orientation of the empty *dif* module are indicated as in Fig. 2. The * above a p*dif* site indicates that the site may not be functional due to mutation of a conserved base in the XerD binding site. Regions of significant DNA conservation are indicated by gray shading with the DNA identity (%) indicated. The sequence of the p*dif* sites involved in each rearrangement event is shown, spacers that have the same sequence are indicated in orange, purple or green. Drawn to scale from GenBank accession numbers listed in Table 2.

Plasmids p2_005069 and pD1279779 differed by a single module (Fig. 9A). In this case, the *dif* module arrangement was similar, but the *sup-uspA* module in p2_005069 had been replaced by a novel module encoding an Orf of 264 aa in pD1279779. The spacer of the p*dif* site on the left of the different modules differed by a single base in the two plasmids and the spacer on the right was the same. A further group of plasmids shared the backbone and two or three modules with pMSHR_A204 but had acquired additional modules, specifically a D module that has been replaced by a D module and a CD module (Fig. 9B and C); and again, the spacers are identical or differ at a single position found in this group. In the final member of this group, pPM194229_3, a D module plus a CD module has replaced a D module in BSH4 p1 (Fig. 9C). The implications of these observations in relation to how a single *dif* module could relocate are discussed further below.

**Fig 10:**
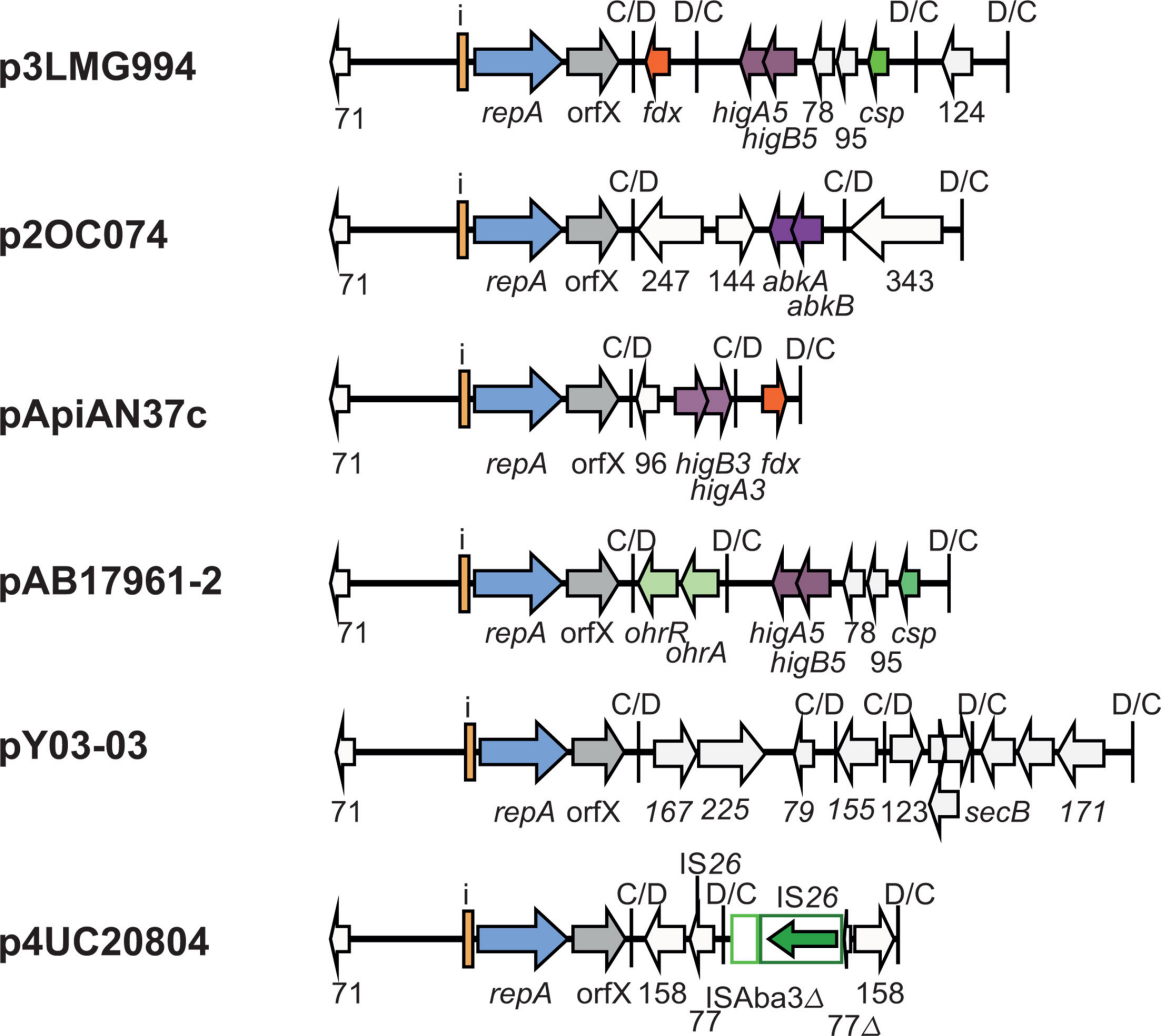
Structure of other r3-T5 non-cointegrate plasmids. The extent and orientation of genes and open reading frames are indicated by arrows colored as in Fig. 2 and 3 with their names below. The *ohrA* and *ohrR* genes, which encode proteins involved in organic peroxide resistance, are light green; *csp*, encoding a cold shock protein, is dark green; and *fdx* is dark orange. The iterons, p*dif* sites, and the orientation of the empty *dif* module are indicated as in Fig. 2. IS*26* and ISAba3Δ are indicated by open green boxes. The Tnp26 transposase gene of IS*26* is shown as a green arrow within the box. Drawn to scale from GenBank accession numbers listed in Table 2.

The remaining non-cointegrate configurations, which include a wide range of accessory modules, are shown in Fig. 10. In this group, there was little overlap with the modules found in pMSHR_A204, and the toxin-antitoxin pair was quite variable (*higAB3*, *higAB5,* or *abkAB*) or, in two cases, absent.

### r3-T5 cointegrate plasmids

**Fig 11:**
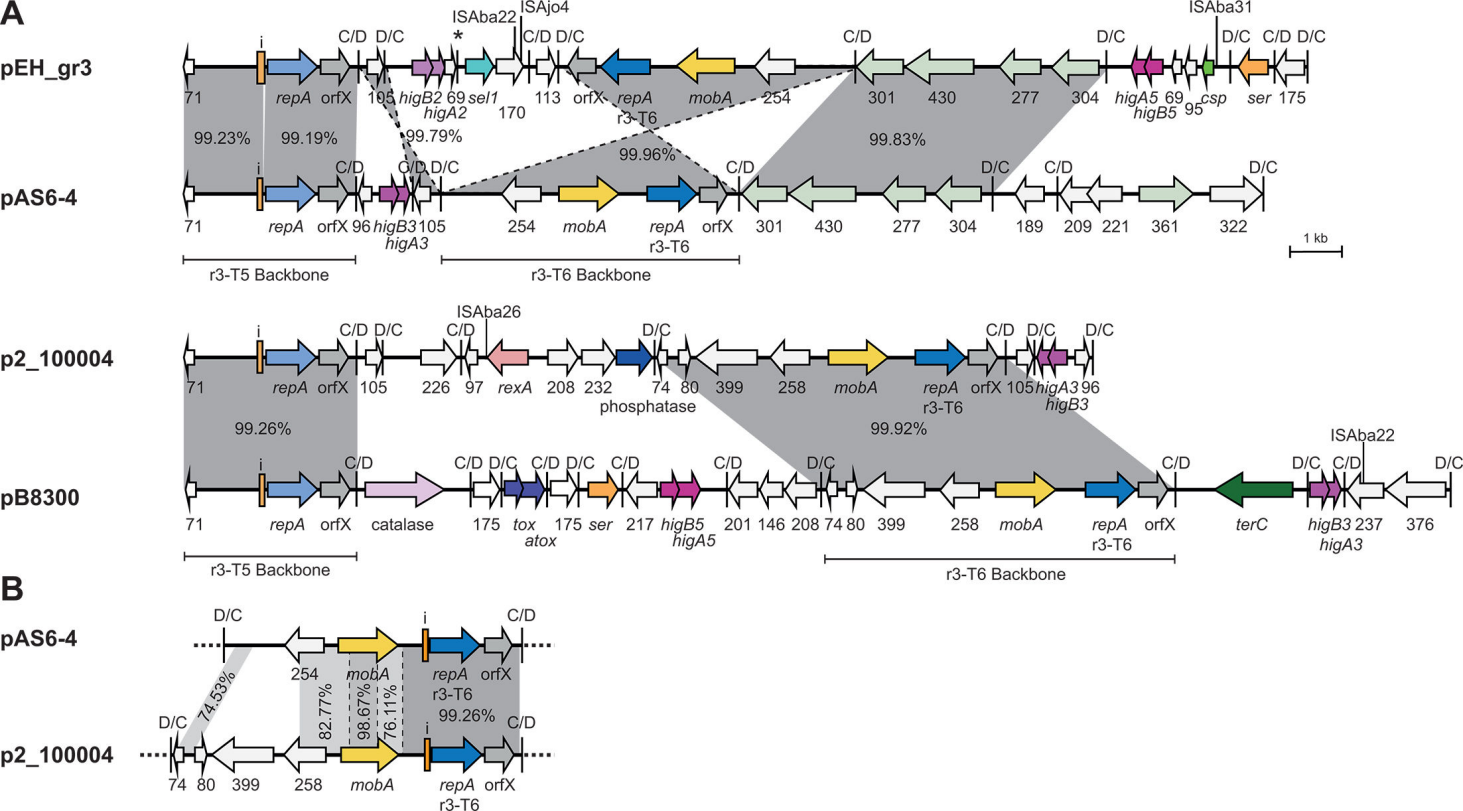
Cointegrate plasmids with r3-T5 and r3-T6 backbones. (**A**) Structure of r3-T5 cointegrate plasmids that have an r3-T6 type second backbone. (**B**) Comparison of two r3-T6 backbone types. The extent and orientation of genes and open reading frames are indicated by arrows with their names below. Genes and open reading frames are colored as in Fig. 2, 4, and 10. Mobilization genes are yellow; *rexA* and catalase genes are different shades of pink; and *terC* is dark green. The type of the second *repA* gene is indicated below the gene name. The iterons and p*dif* sites are indicated as in Fig. 2. The * above a p*dif* site indicates that the site may not be functional. Regions of significant DNA identity are indicated by gray shading, with the DNA identity (%) indicated. Insertion sequence locations are indicated by a vertical bar with the name above. Drawn to scale from GenBank accession numbers listed in Table 2. In A, the location of each backbone module is indicated by a horizontal bar with the type below.

**Fig 12:**
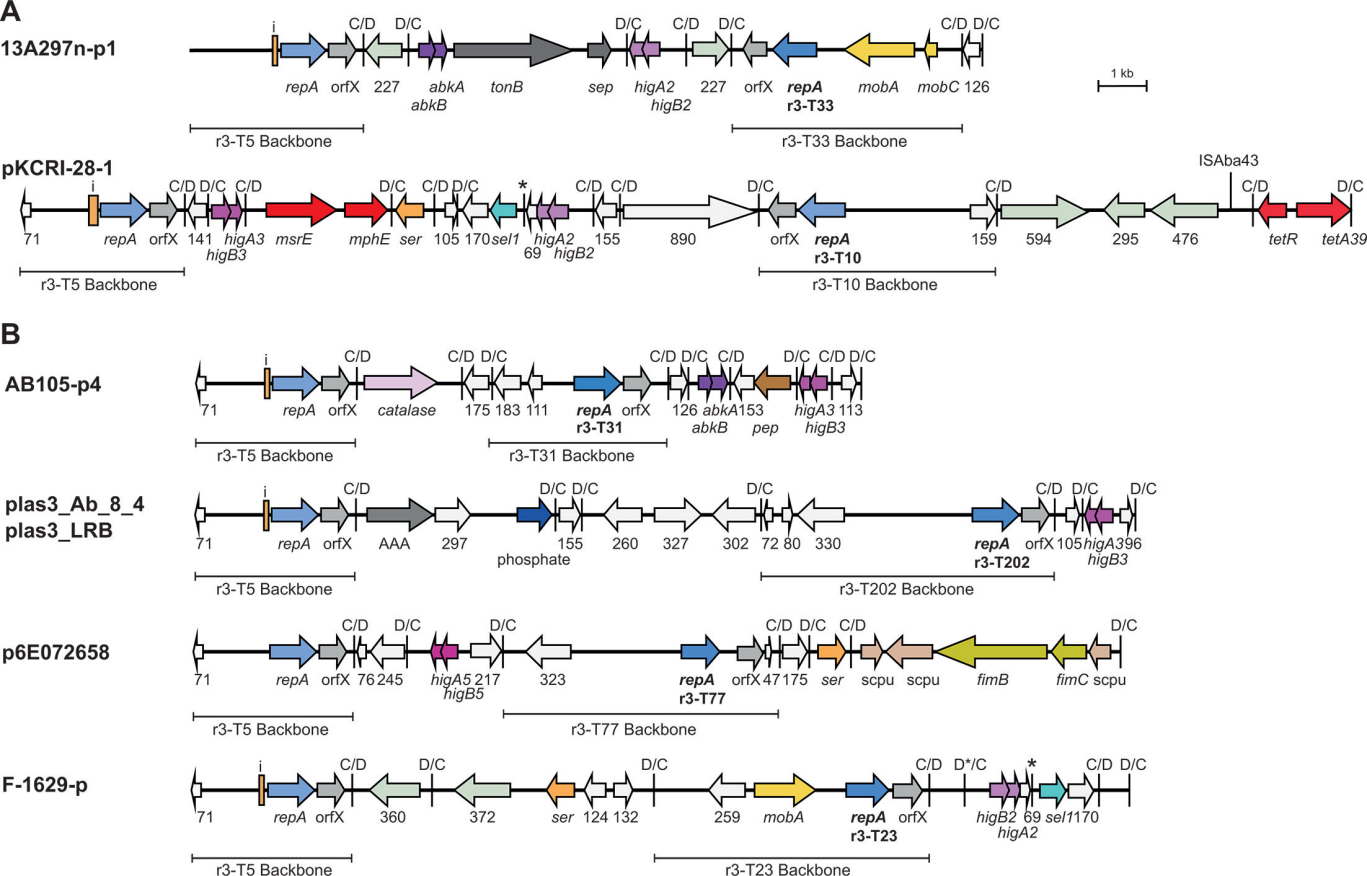
Other r3-T5 cointegrate plasmids. Structure of r3-T5 cointegrate plasmids with a different second backbone type in (**A**) inverse orientation and (**B**) direct orientation relative to the r3-T5 backbone. The extent and orientation of genes and open reading frames are indicated by arrows with their names below. Genes and open reading frames are colored as in Fig. 2 and 11. Mobilization genes are yellow; *tonB* and *sep* (septicolysin) are dark gray; *fimB* and *fimC* are green; and *scpu* (spore coat protein) is beige. The type of the second *repA* gene is indicated below the gene name and the location of each backbone module is indicated by a horizontal bar with the type below. The iterons and p*dif* sites are indicated as in Fig. 2. The * above a p*dif* site indicates that the site may not be functional. Drawn to scale from GenBank accession numbers listed in Table 2.

A second Rep_3/OrfX type backbone C module was present in 10 r3-T5 plasmid types, and eight different backbone types were represented (Table 4). These cointegrate plasmids carry a wide variety of *dif* modules (Fig. 11 and 12). However, only one, pKCRI-28-1, included accessory modules carrying antibiotic resistance genes, namely the *tet39* and *msrE-mphE* modules.

**TABLE 4:** Cointegrate r3-T5 plasmids with a second Rep3/OrfX backbone

Plasmid	*repA* identity (%)^*a*^	No. of iterons	R3-T5 backbone		No. of *dif* modules^*c*^	Second *rep*		Toxin/antitoxin module
			Size (bp)^*b*^	Identity (%)^*a*^		Type	Orientation	
pB8300	99.87	4	3,268	99.72	10	R3-T6	Direct	*higBA3, higBA5*
p2_100004	99.68	4	3,267	99.41	5	R3-T6	Direct	*higBA3*
pEH_gr3	99.82	5	3,288	98.68	7	R3-T6	Inverse	*higBA2*, *higBA5*
pAS6-4	99.58	4	3,267	99.38	5	R3-T6	Direct	*higBA3*
pKCRI-28-1^*d*^	100	7	3,334	98.00	10	R3-T10	Inverse	*higBA2*, *higBA3*
F-1629-p	97.95	4	3,274	nd^*e*^	5	R3-T23	Direct	*higBA2*
AB105-p4	99.89	4	3,268	99.75	7	R3-T31	Direct	*abkBA*, *higBA3*
13A297n-p1	99.89	4	3,576	nd^*e*^	5	R3-T33	Inverse	*abkBA*, *higBA2*
p6E072658	99.68	5	3,288	98.65	5	R3-T77	Direct	*higBA5*
plas3_Ab_8_4	99.68	4	3,266	99.38	5	R3-T202	Direct	*higBA3*
plas3_LRB	99.68	4	3,266	99.38	5	R3-T202	Direct	*higBA3*

^*a*^ Nucleotide identity compared to the representative r3-T5 plasmid pABLAC1 (CP007713).

^*b*^ Backbone size includes only one of the adjacent 28 bp p*dif* sites.

^*c*^ The r3-T5 backbone module was not included in the number of *dif* modules. The *higBA2*-*sel1*-orf170 fusion module described previously (3) was counted as a single module.

^*d*^ pKCRI-28-1 carries the *msrE-mphE* and *tet39 dif* modules.

^*e*^ nd, not determined. The identity could not be determined across the complete length of the backbone due to recombination within the backbone.

The two *repA* genes were found in either orientation relative to one another; seven carry them in direct orientation, while three had them in inverse orientation (Fig. 11 and 12). Four plasmids carried an r3-T6 *repA* gene (Fig. 11A), but there appear to be two different backbone C module types that have different additional gene content (compared in Fig. 11B). Among these, pEH_gr3 and pAS6-4 include the same pair of backbone modules, but they are in different orientations. We previously reported that plasmid 13A297n-p1 is a cointegrate, which has both an r3-T33 backbone and an r3-T5 backbone (3), with the two backbones in inverse orientation. Other Rep_3/OrfX types found were r3-T10, r3-T23, r3-T31, r3-T77, and a new r3-T type recently designated r3-T202 (2). The detailed structure of these cointegrates is shown in Fig. 12.

**Fig 13:**
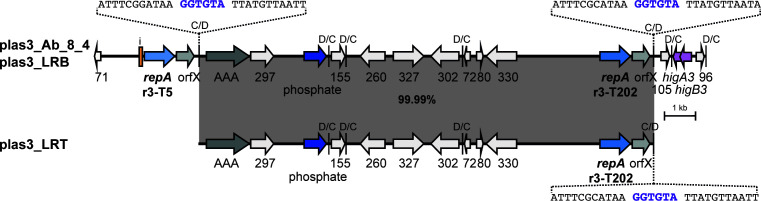
Comparison of the r3-T5, T202 cointegrate plasmid with an r3-T202 simple plasmid. The extent and orientation of genes and open reading frames are indicated by arrows with their names below. Genes and open reading frames of unknown function are colored as in Fig. 2 and 12. The type of the second *repA* gene is indicated below the gene name. The iterons and p*dif* sites are indicated as in Fig. 2. Regions of significant DNA identity are indicated by gray shading, with the DNA percent identity indicated. The sequences of p*dif* sites potentially involved in formation of the cointegrate are shown, with the spacer sequence indicated in purple. plas3_LRT is drawn to scale from GenBank accession number CP121378.

All of these cointegrates likely arose via recombination between p*dif* sites with identical spacer sequences present in the two original plasmids, and, consistent with this, a pair of directly oriented p*dif* sites with identical spacers was detected in 8 of the 10 cointegrate types. In the case of the r3-T5/r3-T202 hybrid plas3_LRB (CP121373) and plas3_Ab_8_4 (CP121368), we found an r3-T202 plasmid (plas3_LRT; GenBank accession number CP121378) that appears to be from the same study, with *dif* modules that correspond to those between the two backbone modules (Fig. 13). Moreover, the spacers in the two sites predicted to have formed the cointegrate are identical, consistent with XerCD-mediated recombination occurring between them.

## DISCUSSION

Plasmids carrying a *repA* gene encoding a Rep_3 family replication initiation protein represent the largest and most diverse group of *Acinetobacter* plasmids, with 200 distinct types identified to date (1, 2). Recently, we showed that almost half of the original 78 r3 types had a C module backbone and carried at least one *dif* module (4), and here we have examined a large set of plasmids belonging to one of these r3 types, namely r3-T5. Members of the T5 group were widely distributed, being found in at least three *Acinetobacter* species and in many different countries. These plasmids shared an identical or very closely related backbone C module but a diverse set of *dif* modules. In several cases, plasmids in the r3-T5 set did not share even one accessory *dif* module (e.g., Fig. 10). However, despite the extensive variation in *dif* module content, in one instance, identical plasmids that were recovered in two different countries and 15 years apart were identified (Table 2), indicating a potential for long-term stability of specific configurations. Only a few plasmids carried a *dif* module encoding an antibiotic resistance determinant, but toxin-antitoxin-encoding modules were abundant and almost universal. The role of these toxin-antitoxin systems remains to be established experimentally, and loss of r3 plasmids is unlikely to occur often, granted that the copy number is generally over five copies per cell, 5.8 for pMSHR-A204 and up to 11 copies in other cases (5, 6, 10, 26), consistent with a good chance of plasmid partition to both daughter cells.

The high backbone conservation observed here for the r3-T5 group is in contrast to the findings of our previous analysis of the r3-T33 plasmid group. In that group, the identity between pairs of *repA* genes ranged from 99.7% to 95.6%, and though the gene content of the backbone C module was conserved in most cases, the *mobC-mobA* region exhibited far greater divergence (up to 15%) than that seen here. In addition, further backbone types were also found among the r3-T33 plasmids (3), and here two backbone module types were found for the r3-T6 backbones found in the cointegrates examined (Fig. 11B). Hence, it appears that an r3-T *repA* type, as currently defined using a cutoff of 5% difference in the *repA* gene sequence, does not always suffice to establish a group of Rep_3/OrfX plasmids with the same backbone C module. This outcome likely arises as a consequence of recombination between backbones of plasmids residing in the same cell. These differences clearly complicate the classification of plasmids, particularly the Rep_3/OrfX types, where in most cases the backbone does not represent the major proportion of the plasmid.

That recombination between p*dif* sites is catalyzed by the XerC and XerD recombinases encoded in *Acinetobacter* chromosomes was recently confirmed experimentally (24). Previous *in vivo* experimental studies have demonstrated both inversion of one or more *dif* modules and loss of pairs of *dif* modules from various Rep_3/OrfX plasmids, and in each case where recombination between a pair of p*dif* sites was detected, the 6 bp spacer that separates the XerC binding site from the XerD binding site has been identical (3, 6, 36). The p*dif* sites involved in formation and resolution of a cointegrate were also identical (21, 24, 27). Here, we noted two forms of pMSHR_A204 that differ by the inversion of a set of three *dif* modules using draft genome sequence reads with the minority form present in 8% of reads spanning the junctions. Hence, these two forms were present in the cell culture used to extract the DNA for sequencing. This inversion was also detected using PCR followed by amplicon sequencing. As we have previously noticed for inversion and loss of *dif* modules, the spacer between the XerC and XerD binding sites of the p*dif* sites involved in this inversion was identical. However, we also detected an inversion and a deletion event in which the spacers differed at a single position adjacent to the XerC binding site, indicating that some flexibility in the sequence, at least at this position, is possible.

In addition, by determining the sequence of the p*dif* sites at the junctions between the backbone C module and the adjacent *dif* module or between pairs of adjacent *dif* modules, we were able to determine that the rearrangements seen in sets of plasmids with the same overall content but different configurations also complied with these rules. Again, in each of the cases we examined, the event giving rise to the observed inversion involved recombination between two inversely oriented p*dif* sites in which the XerC and XerD binding sites were separated by the same 6 bp spacer. Similar observations have been reported previously (9, 37).

**Fig 14:**
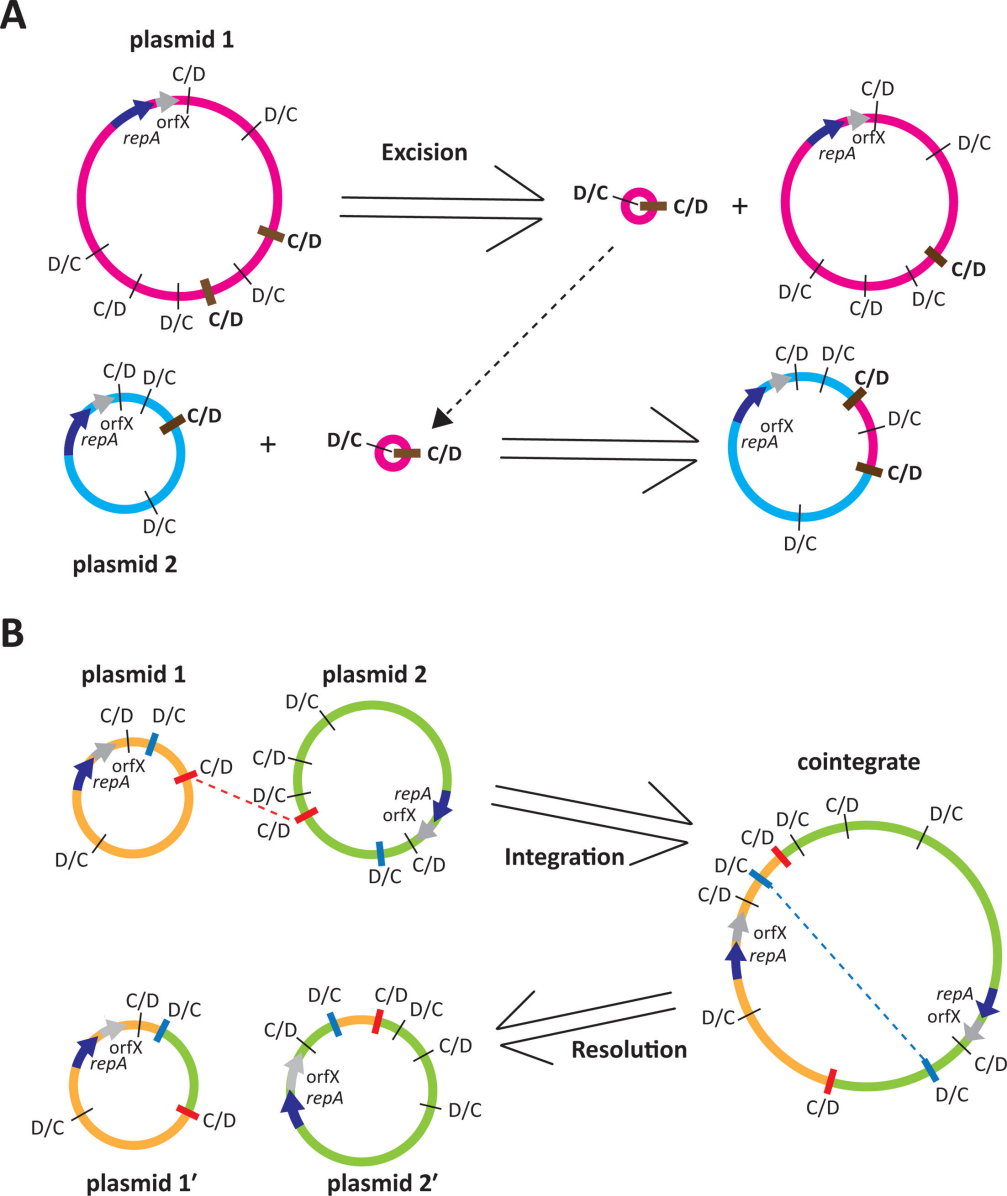
Models for the relocation of *dif* modules. (**A**) Loss or gain of two *dif* modules via excision or integration. (**B**) Exchange of a single *dif* module via cointegrate formation and resolution. Plasmids are indicated by pink, blue, green, or yellow circles with the extent and orientation of *repA* and orfX indicated by a blue or gray arrow, respectively. p*dif* sites are indicated by black bars with the relative orientation (C/D or D/C) indicated. p*dif* sites with the same spacer that are involved in recombination are indicated with thick brown, red, or blue bars. Following integration/excision and cointegrate formation/resolution events, the colors of plasmid segments indicate their origin in the original plasmid configurations.

We examined our findings in the light of the two models for the movement of *dif* modules that have been proposed (5, 20). Both scenarios involve two steps, and both steps would require the availability of p*dif* sites with identical or nearly identical spacer sequences. We originally suggested that pairs of adjacent *dif* modules could be excised by XerCD from one plasmid and then integrated into a different plasmid (5), and both here and previously (6), loss of two different *dif* module pairs was detected experimentally. However, while this route (shown in Fig. 14A) readily explains the movement of an adjacent pair of modules consisting of a standard C and a standard D module to a new location, it cannot explain the movement of a single C or D *dif* module, as each of these is surrounded by inversely oriented p*dif* sites, precluding excision. The alternate route for *dif* module relocation shown in Fig. 14B is via XerCD-mediated formation of a cointegrate followed by XerCD-mediated resolution using a different pair of p*dif* sites (20). In this case, the excision and integration steps occur in a different order, and the likelihood that the integration step proceeds would be greater granted that both participating molecules can replicate. The high proportion of R3-T5 plasmids that were found to be part of a cointegrate indicates that cointegrates are readily formed from two compatible plasmids present in the same cell, and evidence for formation of a cointegrate between two plasmids that coexist in the same cell has been reported (24, 27). Cointegrate resolution has also been demonstrated (21, 27) and the excision of a region bounded by identical, directly oriented *dif*-like sites, which is formally equivalent, has also been demonstrated in *Escherichia coli* (38) and in *A. baumannii* (36) as a method for removal of an introduced selectable marker. However, again, in order to ensure that the alternation of C/D and D/C sites is maintained, modules can only be gained (or lost) in pairs.

In the comparison of p2_005069 and pD1279779 noted above (Fig. 9A) and in the case of the R3-T1 plasmids (7), single *dif* modules had replaced one another, and the spacers in the p*dif* sites on the left side of the pair are near identical, as are those on the right side. However, for these single *dif* modules to be replaced via a cointegrate intermediate, the plasmids forming the cointegrate must be compatible. First, recombination between one pair of p*dif* sites with identical spacers forms a cointegrate between the two precursor plasmids. This is then followed by a XerCD-mediated recombination event between the second pair of p*dif* sites with identical spacers on the other side of the *dif* modules, leading to resolution of the cointegrate coupled with migration of the *dif* module located between the two pairs of sites that participate in the recombination events. The same route could be used to exchange larger groups of modules surrounded by inversely oriented p*dif* sites.

Despite the existence of sequences for many different *Acinetobacter* plasmids, especially as outputs of genome sequencing efforts, very little attention has been directed toward the annotation, comparison, and understanding of the features of these many plasmids. The in-depth analysis undertaken here clearly demonstrates the benefits of such efforts, in this instance contributing to a better understanding of the XerCD-catalyzed *dif* module mobile element system. However, further experimental work could confirm the proposed module movement route.

## MATERIALS AND METHODS

### Bacterial isolates

MSHR_A204 is an ST10:KL108:OCL2 *A. baumannii* strain isolated from a patient with community-acquired pneumonia from the Northern Territory, Australia, in 2011 (28). MSHR_A204 carries the *tet39* tetracycline resistance determinant. MSHR_A204 was kindly supplied by Dr. Nicholas Anstey (Menzies School of Health and Medical Research) and has been stored at −80°C.

### Genome assembly

DNA from the strain MSHR_A204 has been sequenced using the Illumina HiSeq platform (28). Short-read data for MSHR_A204 (BioProject PRJNA478282 and BioSample SAMN09517686) are available under SRA accession number SRR7452008. Here, the genome was *de novo* assembled using SPAdes (version 3.13.0). The assembled genome consisted of 64 contigs with a total length of 3,658,478 bp and an N50 of 274 kb. NCBI AMRFinder+ was used to identify acquired antibiotic resistance genes in MSHR_A204.

### MSHR_A204 plasmid assembly and sequence analysis

The *Acinetobacter* plasmid typing tool (1) was used to screen MSHR_A204 for the presence of plasmid *repA* genes. MSHR_A204 was also screened for the presence of other known *Acinetobacter* plasmids, which do not have an identifiable *repA* gene and are therefore not identified by the typing tool, using an in-house database. Primers designed to amplify across p*dif* sites in pMSHR_A204 are listed in Table S1 and were used in various combinations to determine the plasmid configuration. PCRs were performed on plasmid DNA extracted from MSHR_A204 or transformants containing pMSHR_A204 using conditions described previously (6). The PCR products were visualized on a 1% Tris-borate-EDTA (TBE) gel, followed by gel extraction as previously described (6). Amplicons were sequenced using the same primers at the Australian Genome Research Facility.

Plasmid sequences were annotated manually via inspection using BLAST and an in-house database of known gene sequences. For low-match genes, the InterPro database (https://www.ebi.ac.uk/interpro/) was used to analyze encoded protein sequences and identify conserved domains and protein families. p*dif* sites and *dif* modules were identified manually by first searching for the more conserved XerD binding site, as described previously (5). Where a *dif* site was expected but not identified, the sequence was examined to look for degraded sites with base changes compared to standard sites, as described previously (3). The size of a *dif* module was calculated by including only one of the adjacent 28 bp p*dif* sites.

### Plasmid recovery and analysis

MSHR_A204 liquid culture was inoculated into LB broth (1:100) and cultured until the OD_600_ was ~0.5. Cells were harvested by centrifugation, and plasmid DNA was extracted using an alkaline lysis method as described previously (26). Plasmid DNA was resolved by agarose gel electrophoresis alongside plasmid size standards extracted from *E. coli* strain 39R861+ (39). Plasmid DNA was also digested with restriction enzymes BglII or EcoRI-HF according to the manufacturer’s instructions (New England Biolabs). Digested plasmid DNA was resolved by agarose gel electrophoresis with linear size standards as previously described (26).

### Electroporation of AB307-0294

*A. baumannii* AB307-0294, which contains no plasmids (40), was made electrocompetent as described previously (26). Cells were transformed with 100 ng of MSHR_A204 plasmid DNA by electroporation in 0.1 cm cuvettes using the following parameters: 1.8 kV and ~5 ms time constant τ, as described previously (26). Cells were recovered in SOC liquid media (2.5 mM KCl, 10 mM MgCl_2_, 10 mM NaCl, 2% [wt/vol] tryptone, 0.5% [wt/vol] yeast, 0.02% [wt/vol] glucose) and plated on LB agar containing tetracycline (Tc; 10 mg/L) to select for transformants carrying pMSHR_A204. AB307-0294 was also transformed with 100 ng of pRAY* plasmid DNA ([30]; JQ904627) as a transformation control. LB agar containing kanamycin (Km; 20 mg/L) was used to select for transformants carrying pRAY*.

### Analysis of r3-T5 plasmid sequences in GenBank

Plasmid sequences that included a *repA* gene, which encodes an r3-T5 type RepA, were identified by searches of the GenBank non-redundant database (last searched 30 April 2025) with the *repA* gene sequence from the representative r3-T5 plasmid pABLAC1 (CP007713). The complete sequences of plasmids with a *repA* gene with >95% nucleotide identity to the *repA* gene in pABLAC1 were downloaded and curated to remove ones with multiple sequence errors reflected by multiple pseudogenes or an untrimmed assembly, then annotated manually as described for pMSHR_A204. For ease of comparison between plasmids, each plasmid sequence listed in Table 2 was circularized and reopened so that the complete backbone region was in a single piece at the start of the plasmid. Each plasmid sequence was examined for the presence of additional *repA* genes using the *Acinetobacter* plasmid typing tool (1), and for antibiotic resistance genes using NCBI AMRFinder+. Pairwise BLAST searches of plasmid sequences were used for comparison between plasmids and to examine the relationships between plasmids in this group.

## Data Availability

The pMSHR_A204 plasmid sequence has been released under GenBank accession number PP526176.

## SUPPLEMENTAL MATERIAL

Fig. S1 spectrum.03186-25-s0001.pdf Recombination in the backbone of r3-T5 type plasmids.

Table S1 spectrum.03186-25-s0002.docx Primers for pMSHR_A204 used in this study.

ASM does not own the copyrights to Supplemental Material that may be linked to, or accessed through, an article. The authors have granted ASM a non-exclusive, world-wide license to publish the Supplemental Material files. Please contact the corresponding author directly for reuse.
