# Supplementary material for: Diverse dif module content and configurations in the r3-T5 group of Rep_3/OrfX plasmids from Acinetobacter species reveal extensive dif module shuffling
Source: Microbiol Spectr. 2026 Mar 24;14(5):e03186-25. doi: 10.1128/spectrum.03186-25 (PMC13141974; doi:10.1128/spectrum.03186-25)
Supplement: Fig. S1 — Recombination in the backbone of r3-T5 type plasmids. [file spectrum.03186-25-s0001.pdf]

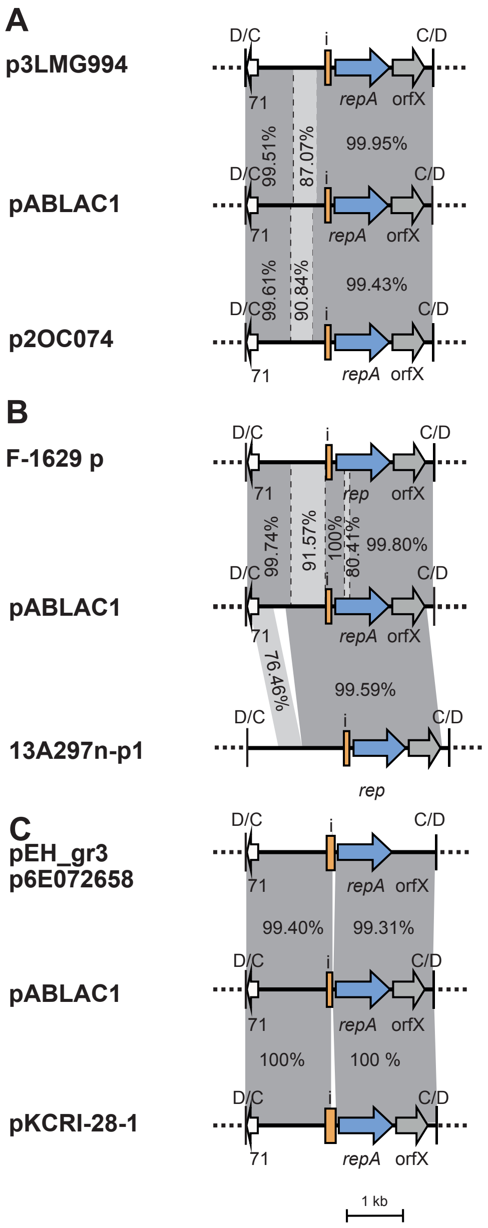

Supplementary Figure 1. Recombination in the backbone of r3-T5 type plasmids. Comparison of (A) p3LMG994 and p2OC074, (B) F1629 p and 13A297n-p1 and (C) pEH_gr3/pE6072658 and pKCRI-28-1 backbone modules with the pABLAC1 backbone module. Arrows indicate the extent and orientation of genes and open reading frames with their names below. The *repA* gene is blue, orfX is grey and reading frames that encode a protein of unknown function are white and are numbered according to the size (aa) of their encoded protein. The iteron region is indicated by an orange box with an i above. p*dif* sites are indicated by a vertical bar with the orientation of the site (C/D or D/C) indicated above. Regions of significant DNA homology are indicated by grey shading with the percent identity indicated. Figures are drawn to scale from GenBank accession numbers in Table 2.
