# Supplementary material for: Diverse dif module content and configurations in the r3-T5 group of Rep_3/OrfX plasmids from Acinetobacter species reveal extensive dif module shuffling
Source: Microbiol Spectr. 2026 Mar 24;14(5):e03186-25. doi: 10.1128/spectrum.03186-25 (PMC13141974; doi:10.1128/spectrum.03186-25)
Supplement: Table S1 — Primers for pMSHR_A204 used in this study. [file spectrum.03186-25-s0002.docx]

**Supplementary Table 1.** Primers for pMSHR_A204 used in this study

| Primer |  | Sequence (5′- 3′) | Position in pMSHR_A204^a^ |
| --- | --- | --- | --- |
| P1 | RH3322 | GAGCATGTTGAACCAGAGCC | 3036-3055 |
| P2 | RH3323 | AGCTCCTGGAATGGTATGGG | 3398-3417 |
| P3 | RH3324 | CGTCCAGTGAAGCAACAGAC | 5952-5971 |
| P4 | RH3325 | TCAGAGTAGGGCATGTGTAAAGT | 6564-6586 |
| P5 | RH3326 | TTGTTGCTCTCTTTCGTCCTG | 6256-6276 |
| P6 | RH3327 | CTGATGGTCATGGTGCCTGA | 7016-7035 |
| P7 | RH3328 | AAGAATACGGACAATGGGGC | 8963-8982 |
| P8 | RH3329 | TCGTCCACTTTTGATACTTAGCC | 9733-9755 |
| P9 | RH3330 | TTCTCGATCAGATGTCTTGCC | 9482-9862 |
| P10 | RH3331 | GCCATGTGCCTTCTGTGAAT | 131-150 |
| *repA*-F | RH3332 | GTTGAAGCAAGGGAATCGGG | 1658-1677 |
| *repA*-R | RH3333 | AAGCGACTTCACTCACCCAT | 1858-1877 |

1. Position in pMSHR_A204-1 nucleotide sequence under GenBank accession number PP526176.
